# Surface improvement of organic photoresists using a near-field-dependent etching method

**DOI:** 10.3762/bjnano.8.81

**Published:** 2017-04-05

**Authors:** Felix J Brandenburg, Tomohiro Okamoto, Hiroshi Saito, Benjamin Leuschel, Olivier Soppera, Takashi Yatsui

**Affiliations:** 1 School of Engineering, University of Tokyo, Bunkyo-ku, Tokyo, 113-8656, Japan; 2 Institut de Sciences des Materiaux de Mulhouse (IS2M), CNRS UMR 7361, 15 rue Jean Starcky, BP 2488, Mulhouse Cedex 68057, France

**Keywords:** near-field etching, organic photoresists, surface improvement, wavelength dependence

## Abstract

Surface flattening techniques are extremely important for the development of future electrical and/or optical devices because carrier-scattering losses due to surface roughness severely limit the performance of nanoscale devices. To address the problem, we have developed a near-field etching technique that provides selective etching of surface protrusions, resulting in an atomically flat surface. To achieve finer control, we examine the importance of the wavelength of the near-field etching laser. Using light sources at wavelengths of 325 and 405 nm, which are beyond the absorption edge of the photoresist (310 nm), we compare the resulting cross-sectional etching volumes. The volumes were larger when 325 nm light was employed, i.e., closer to the absorption edge. Although 405 nm light did not cause structural change in the photoresist, a higher reduction of the surface roughness was observed as compared to the 325 nm light. These results indicate that even wavelengths above 325 nm can cause surface roughness improvements without notably changing the structure of the photoresist.

## Introduction

As the structures fabricated in the field of semiconductors has reached below 10 nm [1], and the pursuit of ever smaller node sizes continues, the impact of the surface roughness (SR) becomes increasingly critical [2]. In attempts to downscale the minimum feature size year-by-year in order to sustain Moore’s Law [3–4], new processing techniques [5] as well as materials [6] are constantly being explored. One important criterion for new materials and techniques is their SR value, and the reduction of the SR is of great interest for high-end lithography.

Conventional chemical mechanical polishing (CMP) methods [7] are generally limited by the roughness of its polishing pad, which is on the order of 10 µm, and by the diameter of the particles in the chemical slurry, which can be around 100 nm. Furthermore, due to issues with cost and material availability, there have been efforts to reduce the usage of the rare-earth material CeO_2_ used in the chemical slurry of CMP [8]. So in order to achieve SR reduction without the use of CMP methods, a novel approach, called near-field etching, has been introduced. This fine-tuning technique has previously proven to be effective in producing atomically flat surfaces in various materials, including GaN [9], glass [10] and even diamond [11] and has shown to be effective on both flat and patterned surfaces.

According to theory, the etching effect originates from radical gas-phase components. More precisely, ambient O_2_ molecules can be dissociated at 5.12 eV [12], and these radical O atoms react with the surface of a specimen. However, it is important to keep in mind that the laser energy used is below 5.12 eV (242 nm) and therefore does not directly cause the dissociation. The etching laser wavelength (325 nm) is carefully chosen to be below the direct O_2_ dissociation energy. Previous theories suggest localized optical near-fields can cause two-step excitation via vibrational levels in molecules [13]. In theory, the localized optical near-field has a nonuniform field distribution and thus can activate the dipole-forbidden intermediate vibrational states of molecules. In other words, although the sample is excited using a laser of energy less than 5.12 eV, owing to the vibrational states, the energy eventually increases to 5.12 eV because of the excitation of localized photons. Furthermore, these localized photons are believed to be primarily present in the protrusions on the surface. Thus, O_2_ dissociation, and hence etching, occurs primarily at locations with a high protrusion density. Previous research suggests that near-field-based energy upconversion can also occur through multiphoton absorption instead of single-photon absorption but the former has a much lower probability [14–15].

The photoresist in this study is a conventional, organic, chemically amplified resist (CAR) (EPIC 2096 ArF Photoresist), which is sensitive to ArF excimer laser excitation (λ = 193 nm). Organic photoresists are easily obtained and play an important role in high-end integrated circuit (IC) chip production. Since the quality of IC semiconductors is directly dependent on their SR, an improvement in the structure of the photoresist during the lithography process could significantly improve the quality of the semiconductor end-product. Furthermore, since near-field etching has only been tested on nonorganic, flat materials and nonpatterned organic materials, it is of great interest to observe the near-field etching effect on organic photoresists.

## Experimental

For the purpose of this study, a positive tone organic photoresist was prepared using interference lithography. Specifically, the chemically amplified resist (EPIC 2096 ArF Photoresist) with an absorption edge of 310 nm was used. To observe the wavelength dependence, the continuous-wave (CW) laser sources used in these experiments were: (1) He–Cd laser (325 nm; 3.81 eV; excitation power density: 0.8 W/cm^2^) and (2) GaN laser (405 nm; 3.06 eV; excitation power: 39 mW). These lasers were carefully selected to be below the dissociation energy of O_2_ (5.12 eV) in order to avoid conventional photochemical etching through direct O_2_ dissociation. The light intensity for all sources except 325 nm was 10^−4^ times greater than the 325 nm source. Therefore, the emission line (except for 325 nm) was negligibly low in intensity and could not have dissociated the O_2_ molecules. The photoresist was illuminated by the respective lasers for a specific time (0–120 min). For the vacuum experiment, before illumination, the sample was fixed into a glass box connected to a vacuum pump. Through a purification process, the O_2_ density inside the glass box was reduced to approximately tens of ppb by first removing the air with the vacuum pump and then refilling the box with N_2_ (containing O_2_ at 5 ppb) until the starting pressure was restored. This process was repeated 10 times, resulting in a low-oxygen (room pressure) environment. The sample surfaces were evaluated using atomic force microscopy (AFM) (Hitachi-Hitech-Science Corp.). The scanned areas were around 3–4 µm in area with a resolution of 256 × 256 pixels. In the AFM software, the “sample intelligent scan” mode was used during the measurement and images were improved using the tilt compensation features. Surface roughness values were automatically obtained by the AFM software, which averaged values of the absolute surface height deviations from a best-fitting plane.

## Results and Discussion

**Figure 1:**
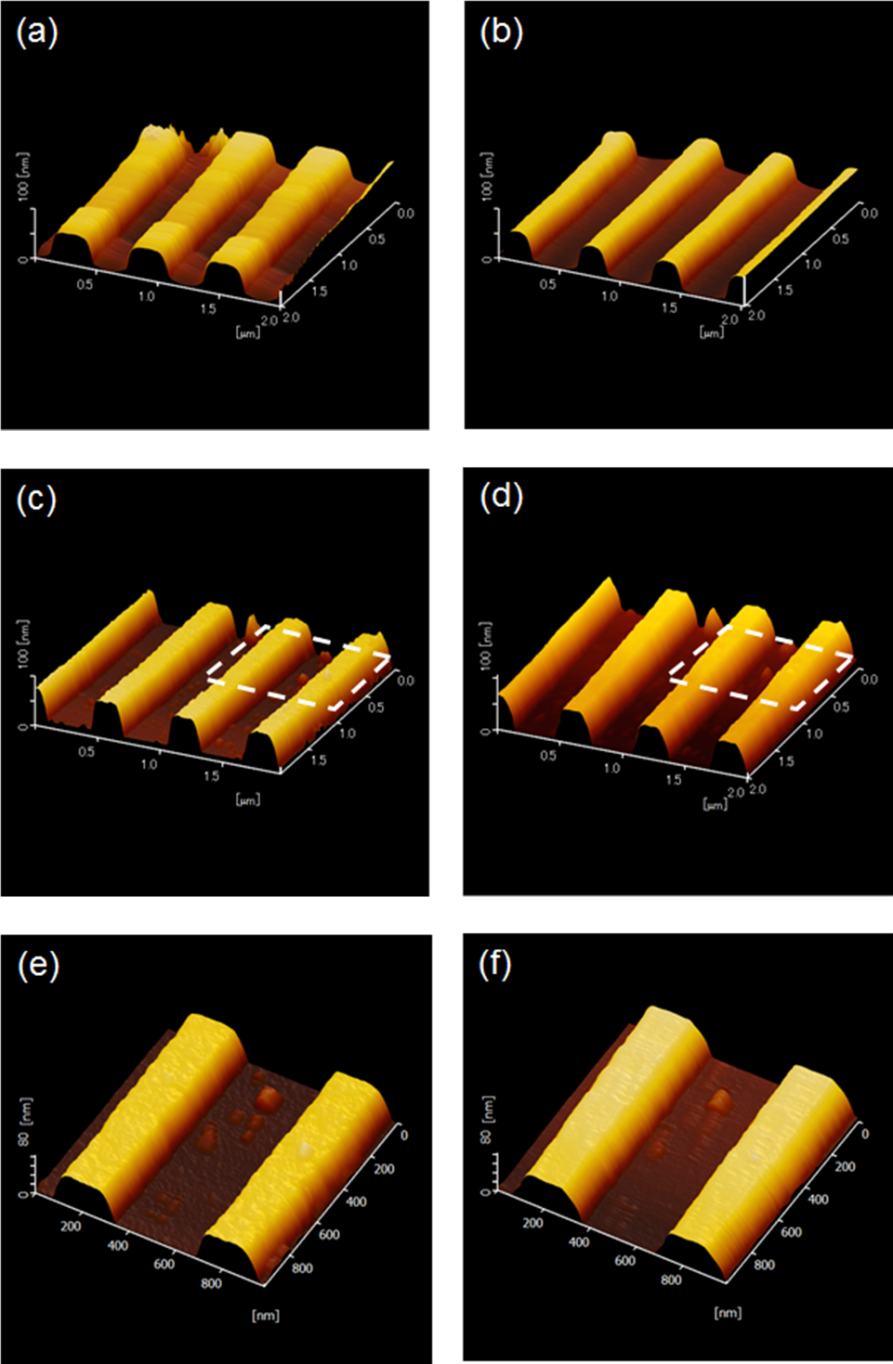
AFM images of the photoresist after 120 min of near-field etching with a He–Cd laser (325 nm, 3.81 eV) ((a) before and (b) after) and a GaN laser (405 nm; 3.06 eV) ((c) before and (d) after). (e) and (f) provide details of (c) and (d) respectively at the same positions (white dashed line).

In order to test the effectiveness of near-field etching, the photoresist substrate was scanned by AFM before and after laser illumination (Figure 1). Figure 1a and Figure 1b show the photoresist before and after near-field etching with a 325 nm laser, respectively. Figure 1c and Figure 1d show the photoresist before and after near-field etching with a 405 nm laser, respectively. The samples were prepared under ambient room conditions, since O_2_ molecules are believed to be integral to this etching phenomenon. Figure 1a and Figure 1b clearly show that illuminating the photoresist with 325 nm laser light changes the structural shape of the photoresist. The images indicate that the photoresist becomes thinner after being illuminated with 325 nm light. The photoresist exposed to near-field etching with 405 nm light in Figure 1c and Figure 1d does not change its structural shape noticeably; however, the surface texture of the photoresist appears to become smoother after near-field etching when looking at the detailed images in Figure 1e and Figure 1f.

**Figure 2:**
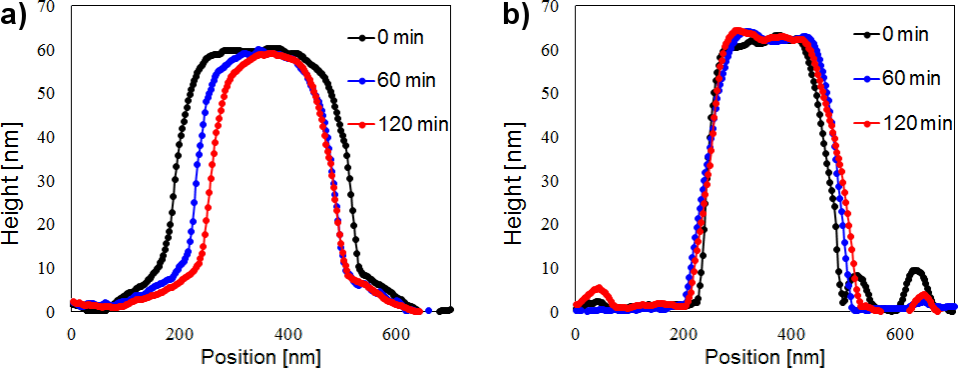
(a) Photoresist profile before (black) and after 60 min (blue) and 120 min (red) of 325 nm laser illumination under room conditions. (b) Same as (a) but with 405 nm laser illumination.

For a more detailed view, Figure 2a illustrates the cross-sectional profile of the photoresist as a function of 325 nm light illumination time. Over this time, the mid-section of the photoresist was reduced from approximately 400 nm to 250 nm in width. In Figure 2b we can see no noticeable change in the cross-sectional profile of the photoresist after near-field etching with 405 nm light. While both laser wavelengths (325 nm and 405 nm) were longer than the O_2_ dissociation wavelength (242 nm) (in order to avoid adiabatic etching), the 325 nm laser excitation appeared to have induced an etching reaction, but the 405 nm laser did not. However the results do not exclude an O_2_ dissociation reaction through the 405 nm laser entirely, if we consider the change of surface smoothness between Figure 1c and Figure 1d. Because the energy of the 405 nm laser (3.06 eV) was significantly lower than the dissociation energy of O_2_, it can be assumed that the 405 nm-laser-induced dissociation of O_2_ molecules is probably attributable to the phenomenon of multiphoton absorption, which occurs far less frequently. In general, it is important to note that the phenomenon of near-field-caused multiphoton absorption is unrelated to the conventional far-field-caused multiphoton absorption. A photon from the 325 nm laser, on the other hand, triggers the dissociation of O_2_ far more readily because its energy is much higher than that of a photon from the 405 nm laser.

**Figure 3:**
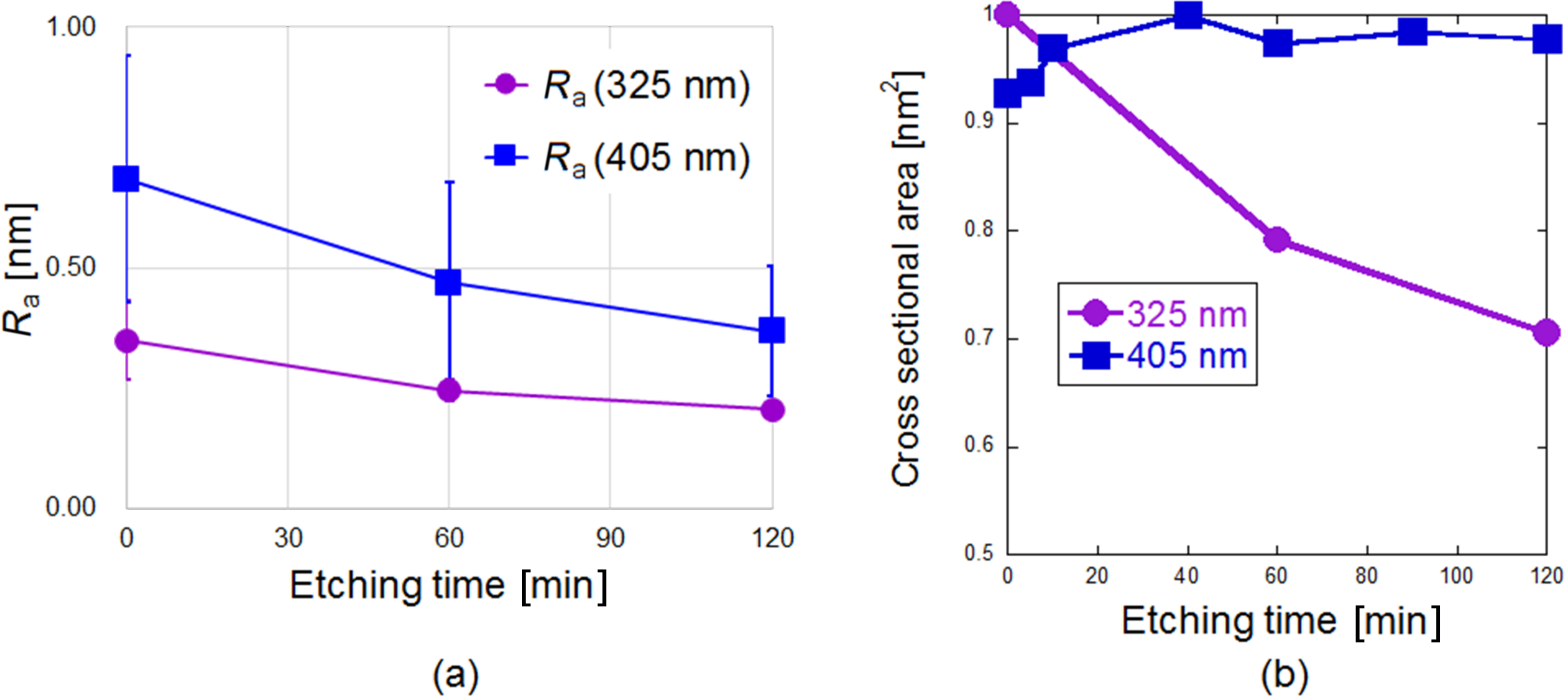
(a) Evaluation of surface roughness reduction of 325 nm (purple) and 405 nm (blue) over a 2 h interval. (b) Showing the cross-sectional etching volume. A comparison of the etching volumes of 325 nm (purple) and 405 nm (blue) over a 2 h interval.

In order to evaluate the degree of surface smoothening by each laser, we evaluated the surface roughness before and after etching through a program embedded in the AFM in Figure 3a. Both lasers were able to smooth the photoresist by reducing its surface roughness over an interval of 2 h. Owing to the nature of the samples, as well as that of the AFM measurement process, fluctuations in the initial SR values were unavoidable. This is the reason for the observed differences in the starting SR values. Although the smoothing rates of the two lasers should not be compared with each other directly, the rate of the 325 nm laser was slightly lower than that of the 405 nm laser. In the case of the 405 nm laser, the difference might be caused by a stronger presence of optical near-fields, owing to the larger initial SR value of the corresponding sample. However, another possible reason could be that the 325 nm laser, through its high etching rate, is removing the surface layers rather than smoothing them (rough versus fine etching), as can be seen in Figure 3b. The 405 nm laser, on the other hand, does not seem to remove any layers of the photoresist. It is mostly smoothing the same outermost layer, hence resulting in a higher surface roughness reduction rate. Again, here we must differentiate between the surface roughness reduction or smoothing rate (Figure 3a) and the gross etching rate (Figure 3b), and how each laser wavelength appears to have comparatively different rates for them.

Furthermore, the results in Figure 2 and Figure 3 indicate that the etching effect of near-field etching is not linear, but actually diminishes over time. Possible explanations for this are that the near-field etching reduces the surface roughness, meaning that after a specific duration there could be less surface protrusions. This seems to be a general condition for near-field etching and is a plausible one since a relative decrease in the number of protrusions during the etching process means a decrease in the generation of the local near-fields as well. The saturation effect has been observed in a wide range of samples and seems to be material independent.

**Figure 4:**
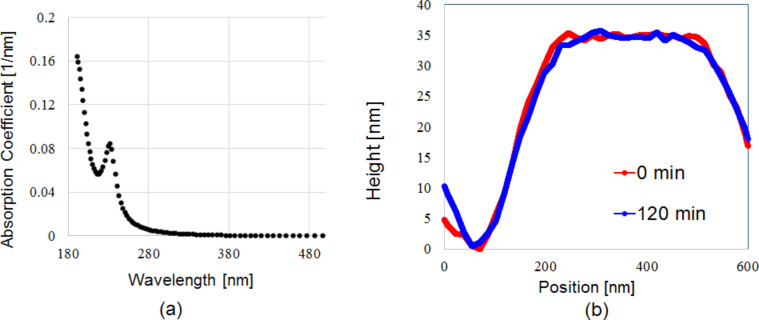
Absorption spectrum and cross-sectional profile. (a) Photoresist absorption curve, showing that 325 nm and 405 nm are not directly being absorbed by the photoresist. (b) Near-field etching for 120 min with 325 nm He–Cd laser, repeated under a low-oxygen environment.

In order to prove that the structural changes are not caused directly by the inherent UV-light sensitivity of the EPIC 2096 ArF photoresist, an absorption spectrum measurement was performed under ambient room conditions (Figure 4a). The spectrum shows that there is no absorption of 325 or 405 nm light. Hence, the inherent UV-light sensitivity of the photoresist can be disregarded as a factor in this paper. Additionally, the same etching experiment (as in Figure 2a) was repeated under a low-oxygen environment, with the result depicted in Figure 4b. Under these conditions, it was not possible to replicate the width loss in Figure 2a. This gives a further reason to assume that radical O atoms are the general cause of the etching phenomena, as well as ruling out the possibility of inherit absorption effects of the photoresist in the case of the 325 nm laser, as shown in Figure 4a. To sum up, the data in Figure 4a and Figure 4b indicates that the etching phenomena is somewhat dependent on ambient O_2_ molecules and that the structural change is not due to the natural UV sensitivity of the photoresist.

## Conclusion

It was possible to change the shape of the photoresist by near-field etching, although the etching ratio is shown to diminish over the etching time. Here, we show that 325 nm illumination changes the cross-sectional width of the photoresist to a greater extent than 405 nm illumination. Conversely, 405 nm light reduced surface roughness to a greater extent than 325 nm light. Overall, this paper proves that near-field etching, through its noncontact nature, could be critical for the development of future nanoscale devices. Furthermore, the results indicate that there is a wavelength dependence of near-field etching, and it would be interesting to further investigate this dependence by examining a wider range of etching wavelengths.
